# Anti-PD-L1 Antibody Enhances T Cell Immune Responses and Reduces Resistance of Breast Cancer Cells to Radiotherapy

**DOI:** 10.1155/2022/5938688

**Published:** 2022-03-07

**Authors:** Lei-Ming Guo, Gao-Feng Ding, Wen-Cai Xu, Hong Ge, Yue Jiang, Yu-Fei Lu

**Affiliations:** Department of Radiotherapy, Affiliated Cancer Hospital of Zhengzhou University (Henan Cancer Hospital), Zhengzhou 450008, China

## Abstract

Immune escape is a frequent occurrence, which limits the duration of antitumor immune responses to radiotherapy. Here, we aimed to ascertain the roles and underlying mechanisms of programmed death ligand 1 (PD-L1) in tolerance of breast cancer (BC) to radiotherapy. We first quantified microRNA-21 (miR-21) and PD-L1 expression in BC tissues and cells, followed by identification of the interactions between miR-21, PD-L1, and programmed cell death protein 4 (PDCD4). miR-21 knock-in mice were used to construct tumor-bearing models, which were then treated with anti-PD-L1 antibody and irradiation, followed by measurement of tumor growth and tumor immune escape. Finally, we evaluated the synergistic effects of radiotherapy and anti-PD-L1 antibody in vivo. The results showed increased miR-21 expression in BC tissues and cells, which was positively correlated with PD-L1 expression. The treatment with radiotherapy or anti-PD-L1 antibody in the miR-21 knock-in mice diminished tumor weight and volume, along with decreased CD3^+^CD8^+^ positive cells, serum IL-2 and IFN-*γ* levels, and lower PD-L1 expression, but augmented apoptosis of T and BC cells. Moreover, miR-21 significantly augmented PD-L1 expression via PI3K/Akt pathway activation by targeting PDCD4 in BC cells. Thus, radiotherapy and anti-PD-L1 antibody synergistically accelerated the therapeutic effect against BC in mice, thereby implicating a close interplay between radiotherapy, T cells, and the miR-21/PDCD4/PI3K/Akt/PD-L1 axis.

## 1. Introduction

Breast cancer (BC) is the most prevalent malignancy in female populations worldwide and accounts for a quarter of all cancers in women, causing around 500,000 deaths annually [1]. In spite of remarkable advancements in molecular targeted therapy [2], BC remains the predominant contributor to the cancer-related deaths in female populations [3]. Adjuvant whole breast radiotherapy is an important treatment modality in the early stage of BC, lowering the risk of local recurrence and death [4]. However, the application of radiotherapy in BC patients is often limited by the risk of its complications, in particular due to poor lung tissue tolerance, which often requires dose reductions or treatment breaks [5].

microRNAs (miRNAs) exert crucial roles in the development of human diseases, particularly in cancers, by acting as tumor suppressors or oncogenes; these properties have made miRNAs attractive tools and targets for novel therapeutic approaches [6]. Overexpression of miR-21 reduces ovarian cancer cell apoptosis and promotes cell proliferation by decreasing phosphatase and tensin homolog deleted on chromosome 10 (PTEN) expression and enhancing phosphatidylinositol 3-kinase/protein kinase B (PI3K/Akt) activity [7]. In addition, miR-21 can potentiate hepatocellular carcinoma cell proliferation and migration while inhibiting apoptosis, by downregulating the TETs/PTENp1/PTEN pathway [8]. High miR-21 expression is linked to lower overall survival time among patients with BC [9]. Notably, miR-21 overexpression is linked to the onset of drug resistance in BC [10].

A recent study indicated that programmed death ligand 1 (PD-L1) protein expression was regulated by miR-21 in gastric cancer [11]. In a previous study of response biomarkers, radiotherapy at a low dose was found to trigger upregulated PD-L1 in tumor cells in various syngeneic models of cancer in mice [12]. BC tissues are frequently infiltrated by inflammatory cells, especially T lymphocytes and macrophages, and T lymphocytes have antitumor activity which can benefit patients' survival [13]. T cell immune responses have the potential to drive epithelial to mesenchymal transition in vivo, thereby producing BC stem cells [14]. PD-L1 has been documented as a critical immune checkpoint molecule that can enable escape of tumor cells from the host immune response via induction of T cell exhaustion and inhibition of effector T cell function [15]. Thus, prolonged treatment with anti-PD-L1 antibody results in persistent tumor regression in 6-17% of cases, together with prolonged stabilization of disease progression manifested in 12-41% cases of patients suffering from advanced BC [16]. In addition, Avelumab, an anti-PD-L1 antibody, presents with an acceptable safety profile and clinical activity in patients with locally advanced or metastatic BC; high PD-L1 expression in tumor-associated immune cells predicts an elevated possibility of clinical response to Avelumab in metastatic BC [17]. Furthermore, radiotherapy potentially provokes a local inflammatory response, thus simultaneously upregulating PD-L1 in the tumor microenvironment, which consequently represses sensitivity to antitumor immune responses [18]. Hence, if it were possible to avoid PD-L1-triggered immune escape, radiotherapy might induce longer lasting control of tumor cells. In this context, the current research intended to delineate the possible effects and elucidate the underlying mechanisms of anti-PD-L1 antibody involvement in T cell immune responses and subsequent radiotherapy tolerance of BC cells.

## 2. Materials and Methods

### 2.1. Ethics Statement

The Ethics Committee of the Affiliated Cancer Hospital of Zhengzhou University (Henan Cancer Hospital) approved the current study protocol, which was in accordance with the Declaration of Helsinki. Written informed consent was provided by each participant prior to sample collection. The animal experiment protocols were approved by the Animal Ethic Committee of Affiliated Cancer Hospital of Zhengzhou University (Henan Cancer Hospital) and were strictly aligned with the Guide for the Care and Use of Laboratory Animal by the US National Institutes of Health.

### 2.2. Bioinformatics Analysis

The BC-related miRNA expression dataset GSE44124, which contained 50 BC tissue samples and 3 normal breast tissue samples, was obtained from Gene Expression Omnibus database. The two groups of data in the microarray were subjected to differential expression analysis with ∣logFoldChange | >1 and *p* < 0.05 as the screening criteria. The downstream regulatory genes of target miRNA were predicted using PicTar, miRDB, TargetScan, and starBase, and the intersection of the four prediction results was obtained. The STRING database was employed to perform the protein-protein interaction analysis, and Cytoscape software was utilized for network visualization.

### 2.3. Study Subjects

We collected 50 clinical samples from BC patients at an early stage (aged 32-65 years with a median age of 50 years) who underwent treatment at the Affiliated Cancer Hospital of Zhengzhou University (Henan Cancer Hospital) between June 2015 and December 2016. Based on the international Tumor Node Metastasis clinical staging of BC [19], 5 tumor tissue samples were at stage I, 33 cases at stage II, and 12 cases at stage III. All patients received breast-conserving surgery after diagnosis by imaging examination and biopsy and were confirmed as invasive ductal breast cancer by postoperative pathology. Subsequent to formalin fixing and paraffin embedding, BC tissues and adjacent normal tissues were preserved at -80°C for follow-up experiments.

### 2.4. Cell Treatment

BC cell lines (BT-20 and MCF-7 [the cell bank of the Chinese Academy of Sciences] and MDA-MB-361 [American Type Culture Collection, ATCC, Manassas, VA, USA]) and human normal breast epithelial cell line MCF-10A (ATCC) were cultured in Dulbecco's Modified Eagle's Medium (DMEM) encompassing 20% fetal calf serum (Invitrogen, Carlsbad, CA, USA) and human insulin (10 *μ*g/mL), followed by placement in an incubator at 37°C with 5% CO_2_. On the next day, cell growth was observed, and the medium was renewed every 1-2 days.

LV5-GFP (Addgene, Cambridge, MA, #25999, for gene overexpression) and pSIH1-H1-copGFP (System Biosciences, Mountain View, CA, LV601B-1, for gene silencing) were selected for the construction of lentiviral vectors. The cells were then treated with miR-21 mimic, miR-21 inhibitor, recombination lentivirus vector harboring overexpression- (oe-) programmed cell death protein 4 (PDCD4) alone or plus miR-21 mimic, recombination lentivirus vector harboring small interfering RNA (siRNA) targeting PDCD4 (si-PDCD4) alone or plus AZD6482, or their corresponding controls (control, mimic negative control [NC], inhibitor NC, oe-NC, and si-NC). Transfection was implemented using Lipofectamine 2000 reagents (Thermo Fisher Scientific, Waltham, MA). miR-21 mimic and inhibitor were synthesized by Suzhou GenePharma Co., Ltd. (Suzhou, China). Infection was conducted using the lentivirus, which was packaged with 293T cells. The cells were then cultured in Roswell Park Memorial Institute 1640 complete medium encompassing 10% fetal bovine serum and passaged every other day. Virus (1 × 10^8^ TU/mL) was supplemented to the BC cell lines for infection.

### 2.5. Establishment of Gene Knock-in Mouse Tumor Models In Situ

A total of 50 miR-21 knock-in (miR-21^+/+^) C57/BL6 mice and 20 wild type (WT) mice were acquired from Nanmo Biotechnology Co., Ltd. (Shanghai, China). BT-20 cells (2 × 10^6^) in the logarithmic growth phase were resuspended in 200 *μ*L DMEM and then injected into the unilateral breast fat pad of the mouse abdomen. After the formation of tumors (about 5 days later), mice were randomized to five groups (*n* = 10 mice/group): miR-21^+/+^, radiotherapy + phosphate buffered saline (PBS) + miR-21^+/+^ (miR-21^+/+^ mice receiving radiotherapy and PBS injection), radiotherapy + miR-21^+/+^ (miR-21^+/+^ mice receiving radiotherapy), anti-PD-L1 + miR-21^+/+^ (miR-21^+/+^ mice receiving anti-PD-L1 antibody treatment), and radiotherapy + anti-PD-L1 + miR-21^+/+^ (miR-21^+/+^ mice receiving radiotherapy and anti-PD-L1 antibody treatment). Anti-PD-L1 antibody was administrated at a dose of 10 mg/kg through intraperitoneal injection, twice a week. WT mice were randomized to 2 groups: WT mice without any treatment and WT mice receiving radiotherapy (*n* = 10 mice/group). Specific intervention measures are shown in Table S1.

The size of tumors was assessed every 2 days subsequent to tumorigenesis, followed by calculation of tumor volume using the formula: L × W^2^/2 (L: length, W: width). The tumors were extracted in all groups 25 days following tumorigenesis to compare the tumor volume and weight. Next, tumor-infiltrating lymphocytes (TILs) were isolated with a tumor dissociation kit (Miltenyi Biotec GmbH, Bergisch Gladbach, Germany) on a gentleMACS Dissociator (Miltenyi Biotec GmbH). Gradient Percoll II solution (GE Healthcare, Little Chalfont, Buckinghamshire, UK) was prepared, and TILs were isolated from the tumor suspension using density gradient centrifugation. Then, flow cytometry was utilized to analyze the TILs. Tumor tissues without TIL isolation were embedded in paraffin and sectioned for immunohistochemical examination.

### 2.6. Gamma Irradiation Treatment

In a mouse model, 5 days following injection of BC cells into the abdominal breast fat pad, the tumorigenic area in mice was irradiated with Cs-137 radiator (HWM D-2000, Siemens Erlangen, Germany) at a single dose rate of 0.95 Gy/min, 3 Gy in total. In addition, the tumorigenic area of control mice was exposed to sham irradiation. The mice were fed normally and the tumors were collected at specified time points.

### 2.7. Reverse Transcription Quantitative Polymerase Chain Reaction (RT-qPCR)

Total RNA extraction was implemented utilizing TRIzol (15596026, Invitrogen) and the RNA was employed as a template to generate complementary DNA (cDNA) using a reverse transcription kit (RR047A, Takara, Tokyo, Japan). For miRNA detection, cDNA of PolyA-supplemented miRNA was obtained using a PolyA tailing detection kit (B532451, Sangon, Shanghai, China). With SYBR Premix EX Taq (RR420A, Takara) for loading, RT-qPCR was implemented on an ABI 7500 instrument (Applied Biosystems, Foster City, CA, USA). At least 3 replicate wells were used. The primers of miR-21, PDCD4, and PD-L1 were prepared by Sangon (Table S2). Glyceraldehyde-3-phosphate dehydrogenase (GAPDH) was used as the reference gene to determine the relative expression levels of PDCD4 and PD-L1, and U6 was used as the reference for miR-21 expression. Fold changes were computed with the 2^-△△Ct^ method.

### 2.8. Western Blot Analysis

Total protein from tissues or cells was initially isolated using a high-efficiency radioimmunoprecipitation assay lysis buffer (R0010, Solarbio, Beijing, China). Thereafter, the concentration of the extracted protein was estimated with a bicinchoninic acid kit (20201ES76, YEASEN, Shanghai, China). After separation using sodium dodecyl sulfate-polyacrylamide gel electrophoresis, the protein was blotted onto polyvinylidene fluoride membranes. Following 1-h blocking at ambient temperature using 5% bovine serum albumin, the membranes were probed overnight at 4°C with primary rabbit antihuman antibodies (Abcam, Cambridge, UK) to PDCD4 (ab51495, 1: 100), PD-L1 (ab205921, 1: 50), PI3K (ab32089, 1: 100), phosphorylated (p)-PI3K (ab182651, 1: 100), Akt (ab8805, 1: 50), and p-Akt (ab81283, 1: 500). The following day, the membranes were reprobed with goat antirabbit immunoglobulin G (IgG) (ab205718, 1: 1000, Abcam) labeled by horseradish peroxidase (HRP) at ambient temperature for 1 h. The immunocomplexes on the membrane were developed with VILBER Fusion FX5 (Vilber Lourmat, Limoges, France), followed by the quantification of band intensities using the ImageJ 1.48u software (National Institutes of Health, Bethesda, MA). The relative protein expression was represented by the ratio of the gray value of the target band to that of GAPDH.

### 2.9. Luciferase Assay

We initially constructed reporter plasmids of PmirGLO-PDCD4-WT-3′untranslated region (3′UTR) and PmirGLO-PDCD4-mutant (MUT)-3′UTR (GenePharma, Shanghai, China). Next, the two plasmids were cotransfected with mimic NC and miR-21 mimic into HEK293T cells, and 48-h culture was performed. Then, using the Dual-Luciferase assay kit (D0010, Solarbio), the luciferase activity was measured on a GLomax20/20 Luminometer (E5311, Shaanxi Zhongmei Biotechnology Co., Ltd., Shaanxi, China).

### 2.10. Immunohistochemistry

Subsequent to 10% formaldehyde fixing, the specimens were sliced at 4 *μ*m, dehydrated, and paraffin-embedded. Antigen retrieval was then implemented. The slices were blocked by the addition of normal goat serum working fluid at 37°C for 10 min before 2-h probing with primary antibodies of rabbit antimouse PD-L1 (1: 100, ab48187, Abcam) and rabbit antibody to Ki67 (ab15580, 1: 200, Abcam) at 37°C. Next, the slices were reprobed with HRP-tagged secondary goat antirabbit IgG (ab6721, 1: 1000, Abcam) in a wet box at 37°C for 30 min, followed by 4-min counterstaining with hematoxylin (Shanghai Fusheng Industrial Co., Ltd., Shanghai, China) at ambient temperature. The excess dye solution was flushed away under running water and the slices were blocked 10% glycerol/PBS for microscopic observation. The results were scored independently by two examiners in a double-blind manner [20].

### 2.11. Immunofluorescence Staining

The cover glasses in the culture plate were fixed in 4% paraformaldehyde for 15 min and reacted with 0.5% Triton X-100 (Sangon) for 20 min at ambient temperature. Thereafter, normal goat serum (Solarbio) was supplemented to the glass for 30-min blocking at ambient temperature, followed by removal of the blocking solution. The glass was then reacted with the following primary rabbit antimouse antibodies (Abcam): PD-L1 (ab48187, 1: 100), CD8 (ab91279, 1: 400), Ym1 (ab192029, 1: 200), and Fizz (ab11429, 1: 100) at 4°C. Next, the glass was incubated with Alexa Fluor 647-labeled secondary donkey antirabbit IgG (ab150075, 1: 400, Abcam) in a wet box at 37°C for 1 h. Subsequent to 5-min nuclei staining with 4',6-diamidino-2-phenylindole (BioDee Biotechnology Co., Ltd., Beijing, China) in subdued light, the glass was covered with fluorescence decay-resistant medium and the images were acquired with a fluorescence microscope (Olympus, Tokyo, Japan) for examination.

### 2.12. Flow Cytometry

Antidifferentiation cluster CD3, CD8, and interferon-*γ* (IFN-*γ*) antibodies were used to detect CD3^+^CD8^+^IFN-*γ*^+^ cells by flow cytometry. The cells were incubated for 30 min at 4°C with rat monoclonal antibodies: fluorescein isothiocyanate- (FITC-) conjugated antibodies (Abcam) to CD8 (ab22378, 1: 500) and IFN-*γ* (ab175878, 1: 100) and PE-conjugated antibody to CD3 (ab135372, 1: 200, Abcam). Following the fixing with 1% paraformaldehyde in PBS, cells were analyzed by a FACSCalibur flow cytometer (Becton Dickinson, Franklin Lakes, NJ, USA). Allotype-matched antibodies were adopted to control nonspecific staining subtracted from the specific staining results. At least 10,000 cells per sample were analyzed with the WinMDI 2.8 software (J. Trotter, Scripps Research Institute, La Jolla, CA).

### 2.13. Annexin V/Propidium Iodide (PI) Staining

After 48 h of cell transfection, apoptotic cells were evaluated utilizing an Annexin V-FITC/PI apoptosis detection kit (556547, BD Company, Franklin Lakes, NJ, USA). Apoptosis was then tested at 488 nm excitation wavelength on a flow cytometer (BD Company).

### 2.14. Terminal Deoxynucleotidyl Transferase Deoxyuridine Triphosphate Nick End Labeling (TUNEL) Staining

The apoptotic cells were characterized using an in situ cell death detection kit (Roche, Basel, Switzerland) [21].

### 2.15. Enzyme-Linked Immunosorbent Assay (ELISA)

A 50 mg portion of tissue was placed in a 2.0 mL centrifuge tube and washed with 0.05 mmol/L precooled PBS. Following this, the fluid was replaced with 0.5 mL precooled PBS and the sample was placed in an ice bath for homogenization using a high-speed tissue homogenizer for 2 min. The supernatant was harvested subsequent to 10-min centrifugation at 3000 r/min and 4°C, and interleukin (IL)-2 and IFN-*γ* levels in the supernatant were determined using an ELISA kit (69-50049, Wuhan Moshake Biotechnology Co., Ltd., Wuhan, Hubei, China). Within 3 min, the measurement of absorbance (A) of each well at 450 nm was recorded using a multimode microplate reader (Synergy 2, BioTek Instrument, Inc., Winooski, VT, USA). A standard curve was calculated by linear regression equation for quantitation of samples.

### 2.16. Statistical Analysis

All data were described as mean ± standard deviation and analyzed by SPSS 21.0 statistical software (IBM Corp., Armonk, New York, USA). The paired *t*-test was adopted to compare the data between BC tissues and adjacent normal tissues, whereas unpaired *t* test was employed to compare data between the two groups. One-way analysis of variance (ANOVA) was applied for the comparison of data between multiple groups, followed by Tukey's post hoc tests. Statistical analysis in relation to time-based measurements for tumor volume was made with ANOVA of repeated measures. A value of *p* < 0.05 was considered statistically significant.

## 3. Results

### 3.1. Expression of miR-21 Was Abundant in Patients with BC after Radiotherapy and Positively Correlated with PD-L1 Expression

**Figure 1:**
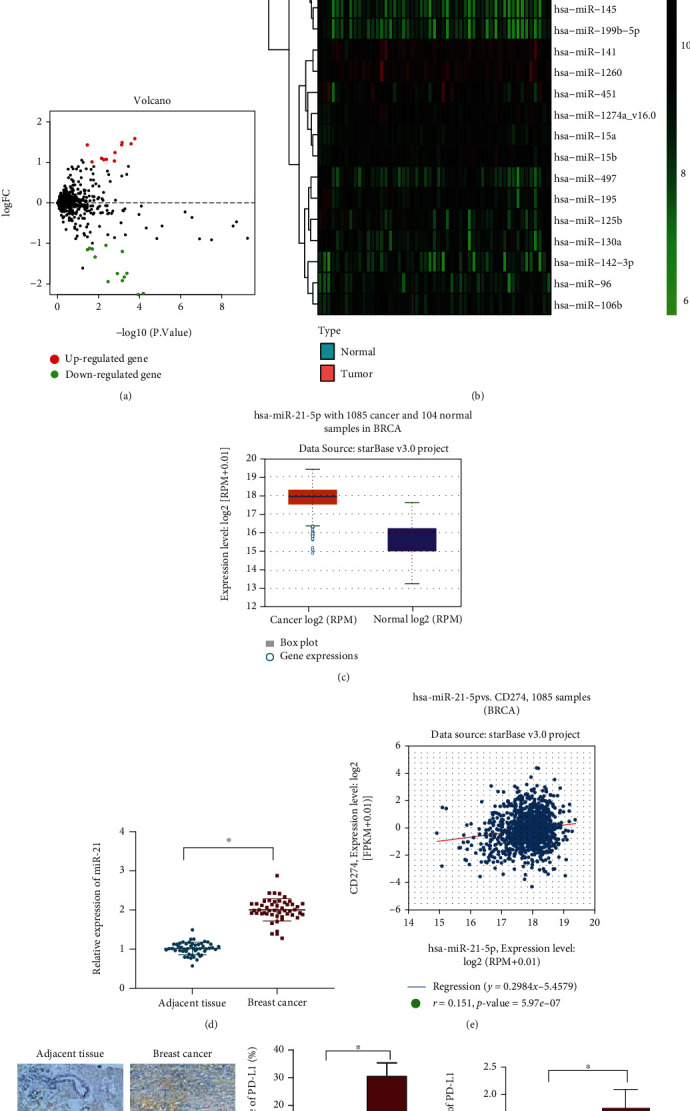
miR-21 expression is increased and positively correlates to PD-L1 expression in BC tissues of patients. (a) Volcano map of differentially expressed miRNAs in 50 BC tissue samples and in 3 adjacent normal tissue samples in the GSE44124 dataset (The *x*-axis represents the difference log10 *p* value and the ordinate represents logFoldChange. Each point in the graph represents a gene, the red point represents the upregulated gene with logFoldChange greater than 1 and the green point represents the downregulated gene with logFoldChange less than -1). (b) Heat map of differentially expressed miRNAs in 50 BC tissue samples and in 3 adjacent normal tissue samples in the GSE44124 dataset. (c) miR-21 differential expression in BC tissue samples and normal breast tissue samples using the TCGA database. (d) Expression of miR-21 in BC and adjacent normal tissues determined by RT-qPCR (*n* = 50). (e) Correlation between PD-L1 and miR-21 expression in BC tissue samples. (f) PD-L1 protein level in BC tissues measured by immunohistochemistry (*n* = 50, scale bar = 50 *μ*m; the nucleus was stained blue, and the positive cells were stained yellow-brown). (g) PD-L1 protein level in BC tissues measured by Western blot analysis (*n* = 50). ^∗^*p* < 0.05 compared with adjacent normal tissues. Data (mean ± standard deviation) from two groups were compared using paired *t*-test or independent sample *t*-test. Each sample was evaluated three times independently.

As tumor suppressors or oncogenes, miRNAs assume a crucial role in the occurrence and development of human diseases, especially malignant tumors [6]. Through differential expression analysis of the BC-related expression dataset GSE44124, 25 differentially expressed miRNAs were obtained, with miR-21 showing the most significant difference (Figures 1(a) and 1(b)). Recent evidence has indicated that miR-21 overexpression inhibits apoptosis of tumor cells and promotes tumor cell proliferation and migration properties by activating the PI3K/Akt pathway [7, 8]. In addition, miR-21 enhances the resistance of BC cells to paclitaxel [10]. Additionally, miR-21 was evident as highly expressed in BC tissues in The Cancer Genome Atlas (TCGA) database (Figure 1(c)). In agreement, RT-qPCR revealed an increased miR-21 expression in BC tissues (Figure 1(d)). Therefore, miR-21 was chosen as the research focus in this study.

A recent study indicates that PD-L1 protein expression is regulated by miR-21 in gastric cancer [11]. Analysis of BC-related datasets in TCGA database illustrated a positive correlation between PD-L1 and miR-21 expression in BC (Figure 1(e)). Moreover, elevated PD-L1 expression was also noted in BC tissues (Figures 1(f) and 1(g), Figure S1A). This indicated that miR-21 was positively correlated with PD-L1, and both of these were highly expressed in BC tissues.

### 3.2. miR-21 Knock-in Enhanced Tolerance of BC Cells to Radiotherapy and Promoted Tumor Immune Escape in Mice

**Figure 2:**
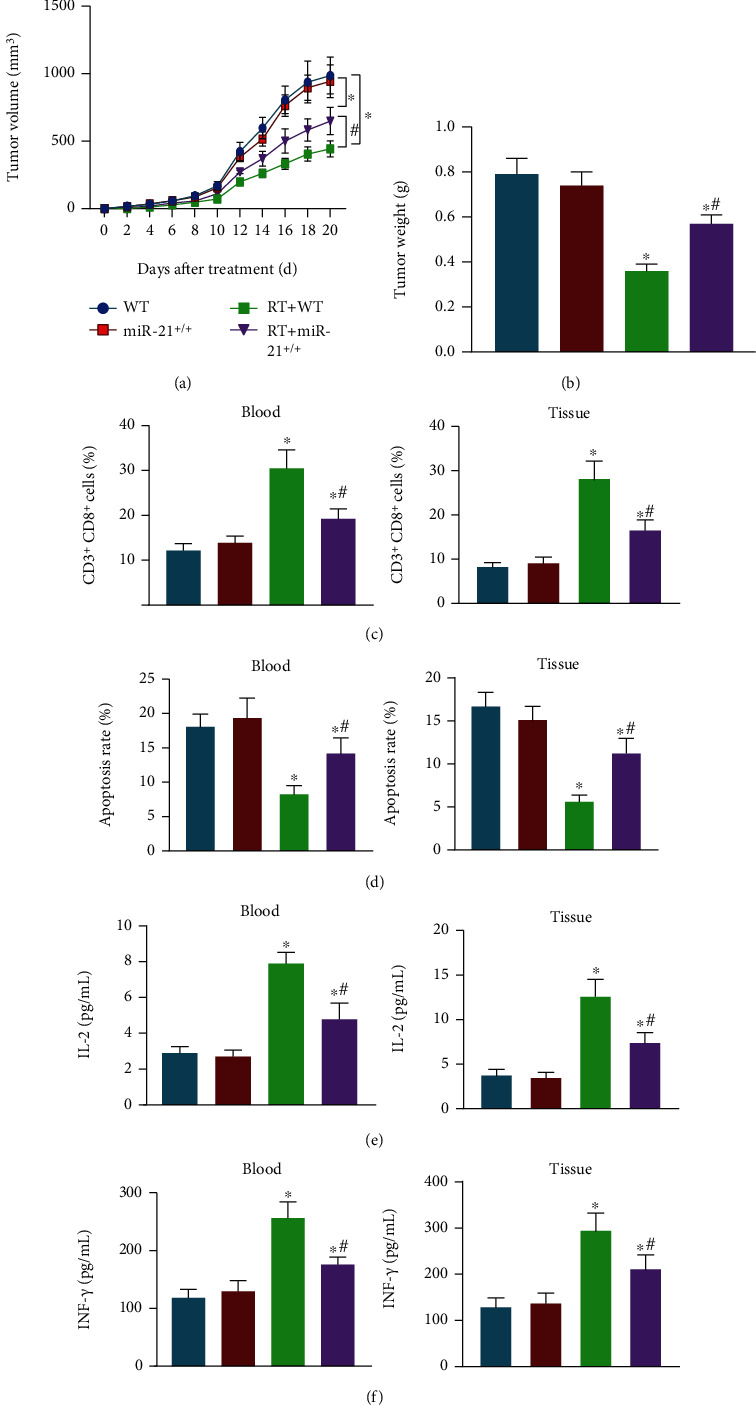
miR-21 knock-in increases tolerance of BC cells to radiotherapy and promotes tumor immune escape in mice. WT or miR-21^+/+^ mice were either treated or not treated with radiotherapy and anti-PD-L1 antibody. (a) Tumor volume curve of the mice. (b) Tumor weight of the mice. (c) The number of CD3^+^CD8^+^ cells in peripheral bloods and tumor tissues of mice detected by flow cytometry. (d) T lymphocyte apoptosis in peripheral blood and tumor tissues of mice measured using Annexin V-FITC/PI. (e) Level of IL-2 in peripheral blood and tumor tissues of mice measured using ELISA. (f) Level of IFN-*γ* in peripheral blood and tumor tissues of mice measured using ELISA. ^∗^*p* < 0.05 compared with WT mice; ^#^*p* < 0.05 compared with WT mice treated with radiotherapy. Each sample was evaluated three times independently. Data (mean ± standard deviation) from two groups were compared using independent sample *t*-test while data for tumor volume were compared using repeated measures ANOVA. There were 10 mice in each group.

To further decipher the influence of miR-21 on BC radiotherapy, miR-21^+/+^ mouse tumor models in situ were established following radiotherapy. The tumor volume and weight of the mice following a total radiotherapy of 3 Gy (0.95 Gy/min) were monitored for 20 days. The results depicted in Figures 2(a) and 2(b) showed that the tumor weight and volume decreased in the WT mice and miR-21^+/+^ mice following radiotherapy as compared with the miR-21^+/+^ mice without treatment. In comparison to the WT mice, the tumor weight and volume of miR-21^+/+^ mice displayed a significant increase under exposure to radiotherapy. These results suggested that the tumors of miR-21^+/+^ mice showed high radiotherapy tolerance.

At the same time, flow cytometry and ELISA documented that the WT mice and miR-21^+/+^ mice following radiotherapy exhibited increased numbers of CD3^+^CD8^+^ positive cells and IL-2 and IFN-*γ* levels and decreased T lymphocyte apoptosis rate in peripheral blood and tumor tissues as compared with the miR-21^+/+^ mice without treatment. The numbers of CD3^+^CD8^+^ positive cells and levels of IL-2 and IFN-*γ* were slightly reduced and T lymphocyte apoptosis rate was slightly elevated in peripheral bloods and tumor tissues of the miR-21^+/+^ mice following radiotherapy in contrast to the WT mice following radiotherapy (Figures 2(c)–2(f)). However, no significant differences were witnessed in these features between WT and miR-21^+/+^ mice without radiotherapy. Collectively, the number of immunocompetent cells decreased in the miR-21^+/+^ mice, which promoted the immune escape of BC cells.

### 3.3. miR-21 Promoted PD-L1 Expression via the PI3K/Akt Pathway Activation by Targeting PDCD4 in BC Cells

**Figure 3:**
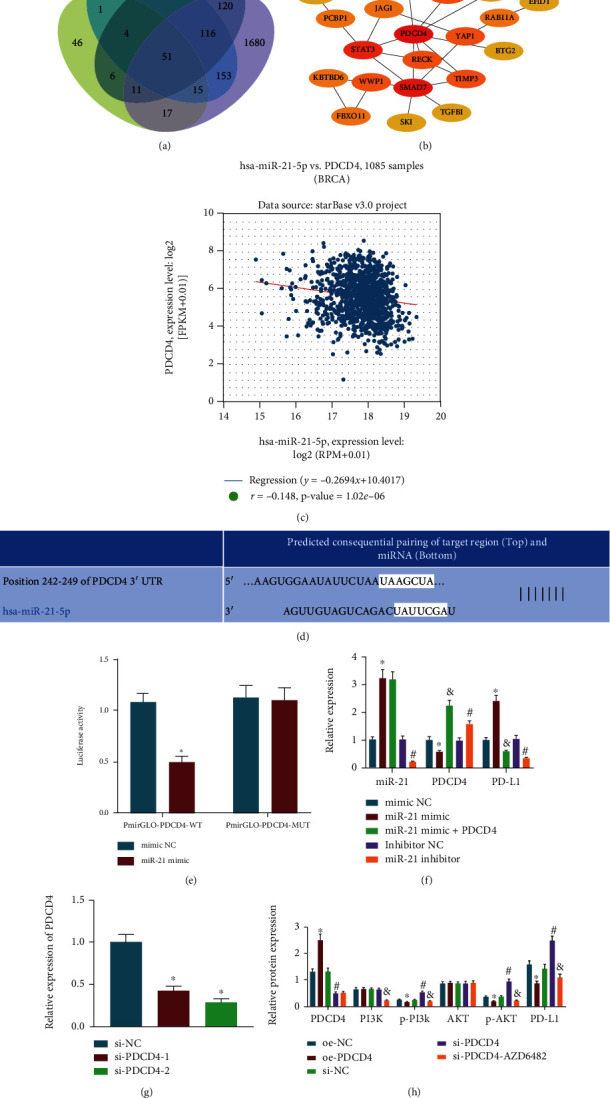
miR-21 potentiates PD-L1 expression via activation of the PI3K/Akt pathway by targeting PDCD4 in BC cells. (a) Venn diagram depicting the intersection of target genes of miR-21 predicted with PicTar, miRDB, TargetScan, and starBase databases. (b) Protein-protein interaction network of the miR-21 downstream target genes. (c) Correlation between PDCD4 and miR-21 expression in BC tissue samples from the TCGA database. (d) Binding site between miR-21 and PDCD4 predicted using the TargetScan database. (e) The binding of miR-21 to PDCD4 confirmed by dual luciferase reporter gene assay. (f) Expression of miR-21, PDCD4, and PD-L1 after alteration of miR-21 and overexpression of PDCD4 determined by RT-qPCR in BC cells. ^∗^*p* < 0.05 compared with cells treated with mimic NC. ^#^*p* < 0.05 compared with cells treated with inhibitor NC and *p* < 0.05 compared with cells treated with miR-21 mimic. (g) The silencing efficiency of si-PDCD4-1 and si-PDCD4-2 in BC cells determined by RT-qPCR. ^∗^*p* < 0.05 compared with cells treated with si-NC. (h) Western blot analysis of PDCD4, PI3K, Akt, and PD-L1 proteins, and the extent of PI3K and Akt phosphorylation in BC cells. ^∗^*p* < 0.05 compared with cells treated with oe-NC. ^#^*p* < 0.05 compared with cells treated with si-NC and *p* < 0.05 compared with cells treated with si-PDCD4. Data (mean ± standard deviation) from two groups were compared using independent sample *t*-test while those from multiple groups were compared using one-way ANOVA. All cell experiments were repeated 3 times independently.

Aiming to dissect the mechanism of miR-21 regulating PD-L1 in BC cells, we initially predicted the target genes of miR-21 via the PicTar, miRDB, TargetScan, and starBase databases. Intersection of the predicted results showed 51 target genes (Figure 3(a)). The protein interaction of 51 candidate target genes was analyzed using STRING database, which displayed that PDCD4 was one of the core genes (Figure 3(b)). In addition, an inverse correlation was noted between PDCD4 and miR-21 expression by analyzing the BC dataset of TCGA database (Figure 3(c)). Previously, miR-21 was found to enhance the progression and facilitate the resistance of BC cells to paclitaxel treatment by targeting PDCD4 [19]. Therefore, PDCD4 was selected as a focus of this research project.

The TargetScan database revealed a binding site between miR-21 and PDCD4 (Figure 3(d)). In addition, luciferase assay manifested that miR-21 mimic diminished the luciferase activity of PmirGLO-PDCD4-WT, but there was no significant difference in the luciferase activity of PmirGLO-PDCD4-MUT (Figure 3(e)), highly suggestive of the targeting relation between miR-21 and PDCD4.

As depicted in Figure S2, compared with MCF-10A cells, miR-21 expression increased, while PDCD4 expression decreased in BC cell lines, and the highest expression of miR-21 and the lowest expression of PDCD4 were found in MCF-7 cells. Thus, MCF-7 cells were selected to further demonstrate the regulatory relationships between miR-21, PDCD4, and PD-L1. As described in Figure 3(f), miR-21 mimic treatment induced elevated expression of miR-21 and PD-L1 yet decreased PDCD4 expression, while miR-21 inhibitor treatment caused opposite effects. In addition, there was a downward trend regarding PD-L1 expression in cells treated with both miR-21 mimic and oe-PDCD4.

It has been found that disruption of the PI3K/Akt pathway can reduce PD-L1 expression and prevent tumor immune escape [20]. To identify whether PDCD4 regulates PD-L1 through the PI3K/Akt pathway, we first constructed siRNAs targeting PDCD4. The silencing efficiency of si-PDCD4-2 was significantly higher and was consequently used for the follow-up experiments, designated as si-PDCD4 (Figure 3(g)). MCF-7 cells were treated with oe-PDCD4, si-PDCD4, or both si-PDCD4 and the PI3K inhibitor AZD6482. There was increased PDCD4 expression and reduced PD-L1 expression and PI3K and Akt phosphorylation in cells upon oe-PDCD4 treatment. However, treatment with si-PDCD4 had the opposite effects. In addition, PI3K and PD-L1 expression together with PI3K and Akt phosphorylation was decreased in cells following both si-PDCD4 and AZD6482 (Figure 3(h), Figure S1B). We further chose an orthotopic xenograft tumor model established using the miR-21^+/+^ mice after radiotherapy to detect differential changes. The results manifested that miR-21 expression and PI3K and Akt phosphorylation levels were augmented, and PDCD4 mRNA and protein expression were diminished in the miR-21^+/+^ mice treated with radiotherapy in contrast to the WT mice treated with radiotherapy (Figure S3). Therefore, miR-21 targets PDCD4 and then induces activation of the PI3K/Akt pathway to upregulate PD-L1 in BC cells.

### 3.4. Anti-PD-L1 Antibody Combined with Radiotherapy Inhibited BC Cell Growth and Promoted Apoptosis In Vivo

Radiotherapy is the conventional treatment of BC, but most patients with BC suffer radiation resistance in the process of treatment [21]. At present, anti-PD-L1 antibody can effectively improve the radiation resistance of tumor cells [22], but there are few studies describing the impact of anti-PD-L1 antibody on the radiation resistance of BC cells.

**Figure 4:**
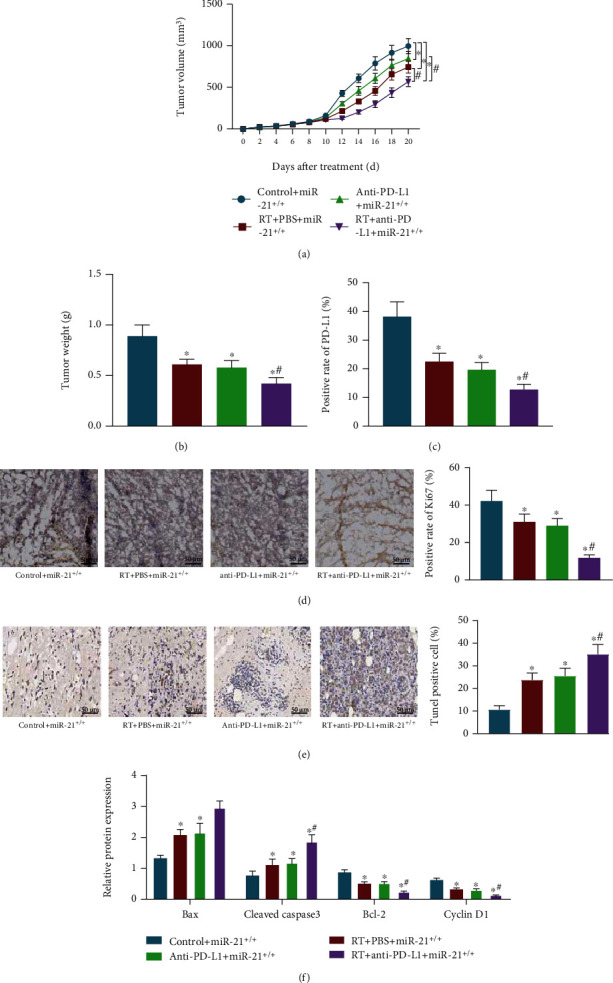
Anti-PD-L1 antibody combined with radiotherapy represses BC cell growth and promotes apoptosis in vivo. miR-21^+/+^ mice were either treated or not treated with radiotherapy or radiotherapy + anti-PD-L1 antibody. (a) Tumor volume curve of miR-21^+/+^ mice following different treatment protocols. (b) Tumor weight of miR-21^+/+^ mice following different treatment protocols. (c) The number of PD-L1 positive cells in tumor tissues of miR-21^+/+^ mice following different treatment protocols, detected by immunofluorescence staining. (d) The positive rate of Ki67 protein expression in tumor tissues of miR-21^+/+^ mice following different treatment protocols, detected by immunohistochemistry (10 mice in each group, 3 sections for each mouse). (e) Cell apoptosis in tumor tissues of miR-21^+/+^ mice following different treatment protocols, measured using TUNEL staining. (f) Protein levels of apoptosis-related factors in tumor tissues of miR-21^+/+^ mice following different treatment protocols, determined by Western blot analysis. ^∗^*p* < 0.05, compared with miR-21^+/+^ mice without treatment; ^#^*p* < 0.05, compared with miR-21^+/+^ mice treated with radiotherapy or anti-PD-L1 antibody. Each sample was evaluated three times independently. Data (mean ± standard deviation) from two groups were compared using independent sample *t*-test. There were 10 mice in each group.

To further explore the role of anti-PD-L1 antibody in BC radiotherapy, miR-21^+/+^ mouse tumor models in situ were established and the BC mice were treated with radiotherapy or anti-PD-L1 antibody. After 20 days of observation, it was found that there was a remarkable reduction in the tumor volume and weight of mice following radiotherapy or anti-PD-L1 antibody alone, which was further reduced by cotreatment of radiotherapy and anti-PD-L1 antibody in miR-21^+/+^ mice (Figures 4(a) and 4(b)).

As depicted in Figure S4A, radiotherapy treatment caused the decline of PD-L1 positive cells in the WT and miR-21^+/+^ mice, while PD-L1 positive cells were enhanced in miR-21^+/+^ mice in contrast to WT mice under exposure to radiotherapy. In comparison to miR-21^+/+^ mice, PD-L1 positive cells (Figure 4(c)) and the positive rate of Ki67 protein expression (Figure 4(d)) were decreased, and apoptosis of BC cells (Figure 4(e)) was increased in the miR-21^+/+^ mice treated with radiotherapy or anti-PD-L1 antibody alone. Radiotherapy combined with anti-PD-L1 antibody further reduced PD-L1 positive cells and the positive rate of Ki67 protein expression and elevated apoptosis of BC cells in the miR-21^+/+^ mice.

Western blot analysis for quantification of apoptosis-related factors also revealed that B-cell lymphoma-2- (Bcl-2-) associated protein X (Bax) and cleaved caspase 3 expression were facilitated, while Bcl-2 and Cyclin D1 expression was restrained by radiotherapy in WT and miR-21^+/+^ mice, while the opposite tendency was witnessed in miR-21^+/+^ mice in comparison to WT mice, in the presence of radiotherapy (Figure S4B). In addition, we also observed elevated protein levels of Bax and cleaved caspase 3 and decreased protein levels of Bcl-2 and Cyclin D1 in the miR-21^+/+^ mice treated with radiotherapy and/or anti-PD-L1 antibody, while more significant changes were detected in miR-21^+/+^ mice after cotreatment of radiotherapy and anti-PD-L1 antibody (Figure 4(f), Figure S1C). Thus, anti-PD-L1 antibody combined with radiotherapy inhibited BC cell growth and promoted apoptosis in vivo to increase the sensitivity of miR-21^+/+^ mice to radiotherapy.

### 3.5. Anti-PD-L1 Antibody Combined with Radiotherapy Suppressed Immune Escape of BC Cells In Vivo

**Figure 5:**
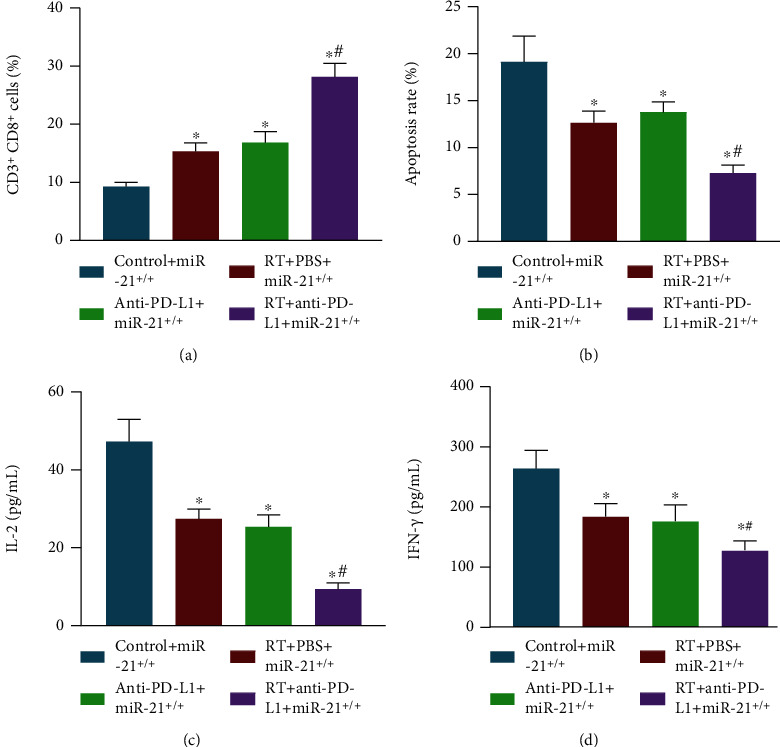
Anti-PD-L1 antibody combined with radiotherapy facilitates T cell immune response in miR-21^+/+^ mice in vivo. miR-21^+/+^ mice were either treated or not treated with radiotherapy or radiotherapy+anti-PD-L1 antibody. (a) The number of CD3^+^CD8^+^ cells in the peripheral blood of miR-21^+/+^ mice following different treatment protocols detected by flow cytometry. (b) T lymphocyte apoptosis rate in peripheral blood of miR-21^+/+^ mice following different treatment protocols measured using Annexin V-FITC/PI. (c) Levels of IL-2 in the peripheral blood of miR-21^+/+^ mice following different treatment protocols measured using ELISA. (d) Levels of IFN-*γ* in peripheral blood of miR-21^+/+^ mice following different treatment protocols measured using ELISA. ^∗^*p* < 0.05 compared with miR-21^+/+^ mice without treatment; ^#^*p* < 0.05 compared with miR-21^+/+^ mice treated with radiotherapy or anti-PD-L1 antibody. Each sample was evaluated three times independently. Data (mean ± standard deviation) from two groups were compared using independent sample *t*-test. The experiments were repeated 3 times independently.

We then determined whether anti-PD-L1 antibody and radiotherapy effectively curtailed T cell immune response in tumor tissues in vivo. Flow cytometry and ELISA exhibited that compared with the miR-21^+/+^ mice without treatment, numbers of CD3^+^CD8^+^ positive cells and levels of IL-2 and IFN-*γ* were elevated, and T lymphocyte apoptosis rate decreased in peripheral blood of miR-21^+/+^ mice following radiotherapy or anti-PD-L1 antibody alone, and the most significant difference was found after cotreatment of radiotherapy and anti-PD-L1 antibody in miR-21^+/+^ mice (Figures 5(a)–5(d)). These results support the notion that cotreatment of anti-PD-L1 antibody with radiotherapy greatly suppressed immune escape of tumor cells in miR-21^+/+^ mice in comparison to anti-PD-L1 antibody treatment or radiotherapy alone, so as to ameliorate radiotherapy resistance of BC cells.

## 4. Discussion

Ionizing irradiation in high doses induces direct tumor cell death and augments tumor-specific immunity, thus enhancing tumor regulation locally and distantly. However, local recurrence frequently occurs following irradiation treatment, which suggests that the irradiation-provoked response is not sufficient to sustain antitumor immunity [18]. Blockade of the T cell negative regulator PD-L1, also known as B7-H1, has the effect of activating T cell effectors in the context of elevated PD-L1 expression occurring in chronically inflamed tissues and tumors [23, 24]. Here, we demonstrated that administration of anti-PD-L1 antibody enhanced the efficacy of radiotherapy through a T cell-dependent mechanism.

We initially found a preferential expression of miR-21 in BC patients following radiotherapy. miR-21 is an oncogene that has been identified to aid in potentiating tumor cell growth and migration abilities in breast, bladder, and colon cancers [25]. Consistent with our present observations, BC tumor samples showed elevated miR-21 expression, and miR-21 expression was transiently upregulated 8 h upon irradiation treatment in radio-resistant T47D cells, and its inhibition attenuated radiation resistance at cellular level [26]. miR-21 has been identified as an independent biomarker of the efficacy of radiotherapy and chemotherapy and thus can be adopted as an effective predictor of response to chemotherapy and radiotherapy in esophageal squamous cell carcinoma [27]. In agreement, the experimental data in our study indicated that miR-21 knock-in enhanced tolerance of BC cells to radiotherapy and promoted tumor immune escape. In partially agreement with our findings, loss of miR-21 was shown to potentiate the polarization of macrophages to an inflammatory M1-like phenotype in tumor cells and thereby confer the host mice with enhanced antitumor immunity [28].

In addition, miR-21 expression has also been observed to be upregulated in colorectal cancer, which results in PD-L1 overexpression through inhibition of PTEN expression [29], suggesting a possible association between miR-21 and PD-L1. Here, we demonstrated that miR-21 expression was positively correlated with PD-L1 expression in BC patients after radiotherapy. miR-21 is implicated in maintenance of the balance between Th17 and Treg cells in patients following gastric cancer resection via the PD-1/PD-L1 pathway [11]. Moreover, PTEN induction has been found to reduce miR-21 expression, thus resulting in downregulation of PD-L1 via the PI3K/Akt/p53 signaling pathway in embryonic cancer stem cells [30]. Accordingly, our experimental results suggested that miR-21 induced PD-L1 expression via the PI3K/Akt pathway activation by targeting PDCD4 in BC cells. Previously, computational analysis indicated that miR-21 can directly target PDCD4 and that the overexpression of miR-21 provokes a reduction in the transcription and protein expression of PDCD4 in cells presenting with the 3′UTR of PDCD4 [31]. miR-21-5p has been demonstrated to augment the progression and paclitaxel resistance in drug-resistant BC cell lines by targeting PDCD4 [19]. In concurrence, the targeting relationship between miR-21 and PDCD4 was detected in our study, while PDCD4 was found poorly expressed in BC cells. PDCD4 overexpression has the capacity to restrict cell growth through its inhibitory role in the PI3K/Akt pathway in non-small-cell lung cancer [32], supporting our findings.

Another central observation in our study was that administration of anti-PD-L1 antibody increased the sensitivity of miR-21^+/+^ mice to radiotherapy. A functional study has demonstrated that activation of the Akt/mammalian target of rapamycin (mTOR) pathway accelerates immune escape by elevating PD-L1 expression, where an mTOR inhibitor in combination with antibody PD-1 reduces tumor growth and regulatory T cells, while increasing the abundance of tumor-infiltrating T cells [33]. Avelumab, an anti-PD-L1 antibody, possesses antitumor property in treating metastatic BC and it has already shown clinical activity and safety in patients suffering from metastatic BC [17]. Notably, upregulated PD-L1 in bladder cancer cells correlates to the irradiation dose, whereas the blockade of PD-L1 delays tumor growth following radiotherapy [34]. Importantly, we verified that anti-PD-L1 combined with radiotherapy improved the therapeutic effect in BC by weakening radiation resistance. Concurrent inhibition of miR-21 in macrophages and anti-PD-1 antibody treatment presented with superior antitumor activity than either agent alone in an earlier study [28]. Of note, radiotherapy combined with immunotherapy is much more effective than monotherapy and has shown promising outcomes in various animal models [35]. Patients with non-small-cell lung cancer receiving combined therapeutic administration of anti-PD-1/PD-L1 antibody and radiotherapy have a significantly better prognosis than those who have not undergone radiotherapy [36]. In the context of invasive BC, radiotherapy combined with anti-PD1/PD-L1 therapy leads to more favorable clinical outcomes among patients [37]. These aforementioned findings provide a rationale for the combination of anti-PD-L1 antibody and radiotherapy in BC.

## 5. Conclusion

**Figure 6:**
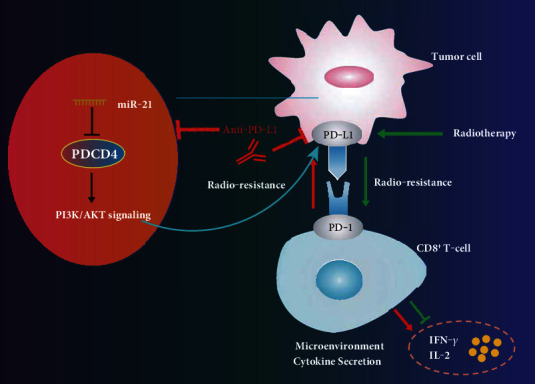
Mechanistic investigations indicated that anti-PD-L1 antibody augmented T cell immune response and alleviated the tolerance of BC to radiotherapy by regulating the miR-21/PDCD4/PI3K/Akt/PD-L1 axis.

Overall, we have demonstrated that anti-PD-L1 antibody treatment can drive T cell immune responses and override the radiotherapy tolerance of BC cells by modulating the miR-21/PDCD4/PI3K/Akt/PD-L1 axis (Figure 6). Our study provides evidence clarifying the effect of anti-PD-L1 antibody as a radiosensitizer. In addition, future studies should be conducted to verify whether miR-21^+/+^ mice might become immunized against the tumor after radiotherapy and anti-PD-L1 treatment and how PI3K is phosphorylated by PDCD4.

## Data Availability

The data and materials of the study can be obtained from the corresponding author upon request.

## Supplementary Materials

Supplementary Materials Figure S1: representative Western blots for panel 1G (A), 3H (B), and 4F (C). A, PD-L1 protein level in BC tissues measured by Western blot analysis (*n* = 50). B, Western blot analysis of PDCD4, PI3K, Akt, and PD-L1 proteins as well as the extent of PI3K and Akt phosphorylation in BC cells. C, Protein levels of apoptosis-related factors in tumor tissues of the miR-21^+/+^ mice determined by Western blot analysis. Figure S2: expression of miR-21 and PDCD4 in BC cells. A, miR-21 expression in human normal mammary epithelial cells (MCF-10A) and BC cells (BT-20, MCF-7, and MDA-MB-361) determined by RT-qPCR. B, PDCD4 expression in human normal mammary epithelial cells (MCF-10A) and BC cells (BT-20, MCF-7, and MDA-MB-361) determined by RT-qPCR. ^∗^*p* < 0.05 compared with MCF-10A cells. Data (mean ± standard deviation) among multiple groups were analyzed using one-way ANOVA. Each sample was evaluated three times independently. Figure S3: miR-21, PDCD4, PI3K, and Akt expression in WT mice and miR-21^+/+^ mice after the treatment with radiotherapy. A, miR-21 expression and PDCD4 mRNA expression in WT mice and miR-21^+/+^ mice after treatment with radiotherapy. B, PDCD4, PI3K, and Akt expression and PI3K and Akt phosphorylation levels in WT mice and miR-21^+/+^ mice after treatment with radiotherapy. ^∗^*p* < 0.05 compared with WT mice treated with radiotherapy. There were 10 mice in each group. Each sample was evaluated three times independently. Figure S4: expression of PD-L1 and apoptosis-related factors in WT mice and miR-21^+/+^ mice treated with radiotherapy. A, PD-L1 positive cells in tumor tissues of WT mice and miR-21^+/+^ mice treated with radiotherapy measured by immunofluorescence. B, Protein levels of apoptosis-related factors in tumor tissues of WT mice and miR-21^+/+^ mice treated with radiotherapy determined by Western blot analysis. ^∗^*p* < 0.05, compared with WT mice treated with radiotherapy. There were 10 mice in each group. Table S1: intervention measures in each group. Table S2: primer sequences for reverse transcription quantitative polymerase chain reaction.
