# Supplementary material for: Anti-PD-L1 Antibody Enhances T Cell Immune Responses and Reduces Resistance of Breast Cancer Cells to Radiotherapy
Source: Oxid Med Cell Longev. 2022 Mar 7;2022:5938688. doi: 10.1155/2022/5938688 (PMC8920704; doi:10.1155/2022/5938688)
Supplement: Supplementary Materials — Figure S1: representative Western blots for panel 1G (A), 3H (B), and 4F (C). A, PD-L1 protein level in BC tissues measured by Western blot analysis (n = 50). B, Western blot analysis of PDCD4, PI3K, Akt, and PD-L1 proteins as well as the extent of PI3K and Akt phosphorylation in BC cells. C, Protein levels of apoptosis-related factors in tumor tissues of the miR-21+/+ mice determined by Western blot analysis. Figure S2: expression of miR-21 and PDCD4 in BC cells. A, miR-21 expression in human normal mammary epithelial cells (MCF-10A) and BC cells (BT-20, MCF-7, and MDA-MB-361) determined by RT-qPCR. B, PDCD4 expression in human normal mammary epithelial cells (MCF-10A) and BC cells (BT-20, MCF-7, and MDA-MB-361) determined by RT-qPCR. ∗p < 0.05 compared with MCF-10A cells. Data (mean ± standard deviation) among multiple groups were analyzed using one-way ANOVA. Each sample was evaluated three times independently. Figure S3: miR-21, PDCD4, PI3K, and Akt expression in WT mice and miR-21+/+ mice after the treatment with radiotherapy. A, miR-21 expression and PDCD4 mRNA expression in WT mice and miR-21+/+ mice after treatment with radiotherapy. B, PDCD4, PI3K, and Akt expression and PI3K and Akt phosphorylation levels in WT mice and miR-21+/+ mice after treatment with radiotherapy. ∗p < 0.05 compared with WT mice treated with radiotherapy. There were 10 mice in each group. Each sample was evaluated three times independently. Figure S4: expression of PD-L1 and apoptosis-related factors in WT mice and miR-21+/+ mice treated with radiotherapy. A, PD-L1 positive cells in tumor tissues of WT mice and miR-21+/+ mice treated with radiotherapy measured by immunofluorescence. B, Protein levels of apoptosis-related factors in tumor tissues of WT mice and miR-21+/+ mice treated with radiotherapy determined by Western blot analysis. ∗p < 0.05, compared with WT mice treated with radiotherapy. There were 10 mice in each group. Table S1: intervention measures in each group. Table S2: [file 5938688.f1.docx]

**TABLE S1** Intervention measures in each group

| Group | Measure | n |
| --- | --- | --- |
| miR-21^+/+^ | Overexpressed miR-21 | 10 |
| RT + miR-21^+/+^ | Overexpressed miR-21 + radiotherapy | 10 |
| RT + PBS + miR-21^+/+^ | Overexpressed miR-21 + radiotherapy and PBS injection | 10 |
| anti-PD-L1 + miR-21^+/+^ | Overexpressed miR-21 + anti-PD-L1 injection | 10 |
| RT + anti-PD-L1 + miR-21^+/+^ | Overexpressed miR-21 + radiotherapy and anti-PD-L1 injection | 10 |
| WT | Untreated WT mice | 10 |
| RT + WT | WT mice treated with radiotherapy | 10 |

Note: miR, microRNA; PBS, phosphate buffered saline; PD-L1, programmed death ligand 1; RT, radiotherapy; WT, wild-type.

**TABLE S2** Primer sequences for reverse transcription quantitative polymerase chain reaction

| Target | Primer sequences |
| --- | --- |
| miR-21 (human) | Forward: 5’-TGCGCTAGCTTATCAGACTGAT-3’ |
|  | Reverse: Universal reverse primer |
| miR-21 (mouse) | Forward: 5’-ATGGTTCGTGGGTAGCTTATCAGACTGA-3’ |
|  | Reverse: Universal reverse primer |
| PDCD4 (human) | Forward: 5’-GTATGATGTGGAGGAGGTGGAT-3’ |
|  | Reverse: 5’-CCCTCCAATGCTA-3’ |
| PD-L1 (mouse) | Forward: 5’-GCTCCAAAGGACTTGTACGTG-3' |
|  | Reverse: 5’-TGATCTGAAGGGCAGCATTTC-3’ |
| U6 (human) | Forward: 5’-CTCGCTTCGGCAGCACA-3' |
|  | Reverse: Universal reverse primer |
| U6 (mouse) | Forward: 5’-CTCGCTTCGGCAGCACATATACT-3’ |
|  | Reverse: Universal reverse primer |
| GAPDH (human) | Forward: 5’-GTCTCCTCTGACTTCAACAGCG-3’ |
|  | Reverse: 5’-ACCACCCTGTTGCTGTAGCCAA-3’ |
| GAPDH (mouse) | Forward: 5’-CAATGAATACGGCTACAGCAAC-3’ |
|  | Reverse: 5’-TGGTCACCAGGGCTGCTTTTA-3’ |

Note: miR, microRNA; PDCD4, programmed cell death protein 4; PD-L1, programmed death ligand 1; GAPDH, glyceraldehyde-3-phosphate dehydrogenase.

**
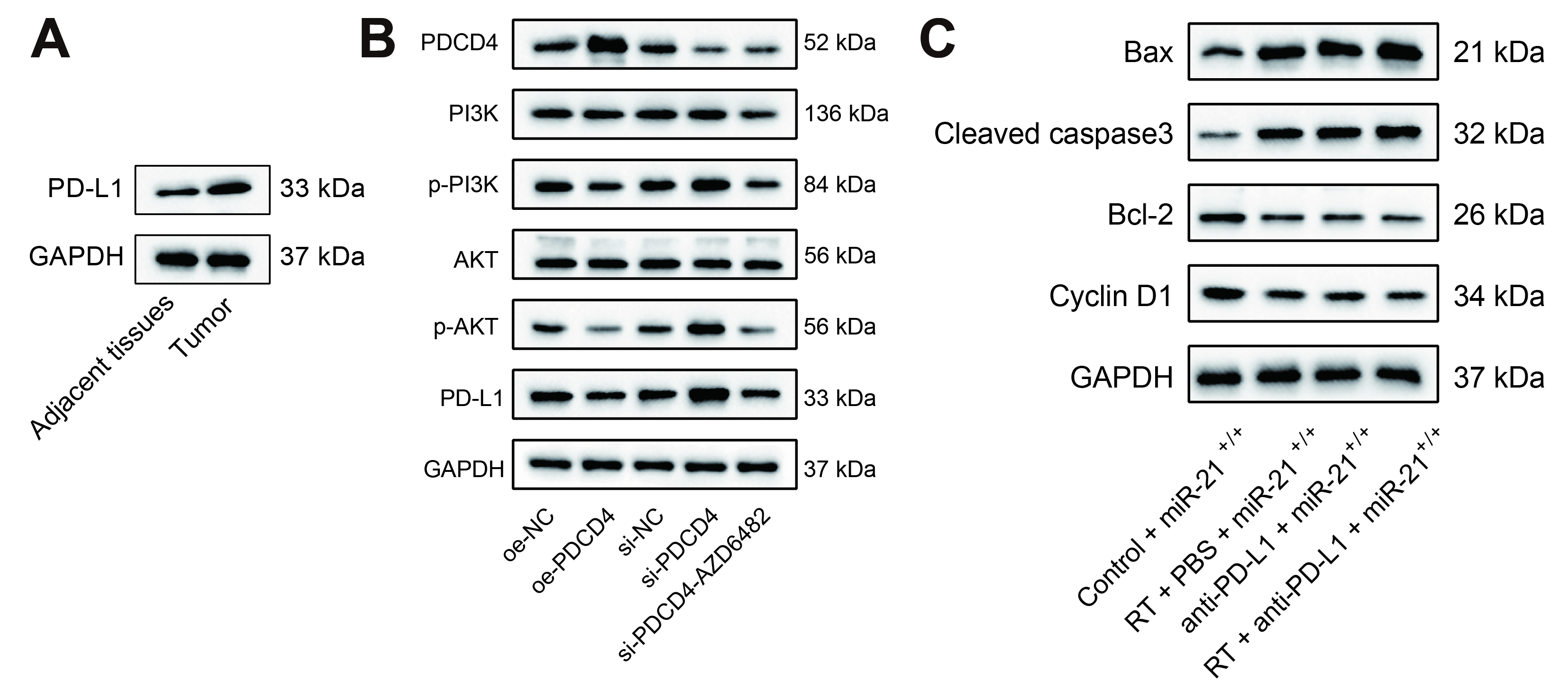
**

**FIGURE S1** Representative Western blots for panel 1G (A), 3H (B), and 4F (C). A, PD-L1 protein level in BC tissues measured by Western blot analysis (n = 50). B, Western blot analysis of PDCD4, PI3K, Akt and PD-L1 proteins as well as the extent of PI3K and Akt phosphorylation in BC cells. C, Protein levels of apoptosis-related factors in tumor tissues of miR-21^+/+^ mice determined by Western blot analysis.

**
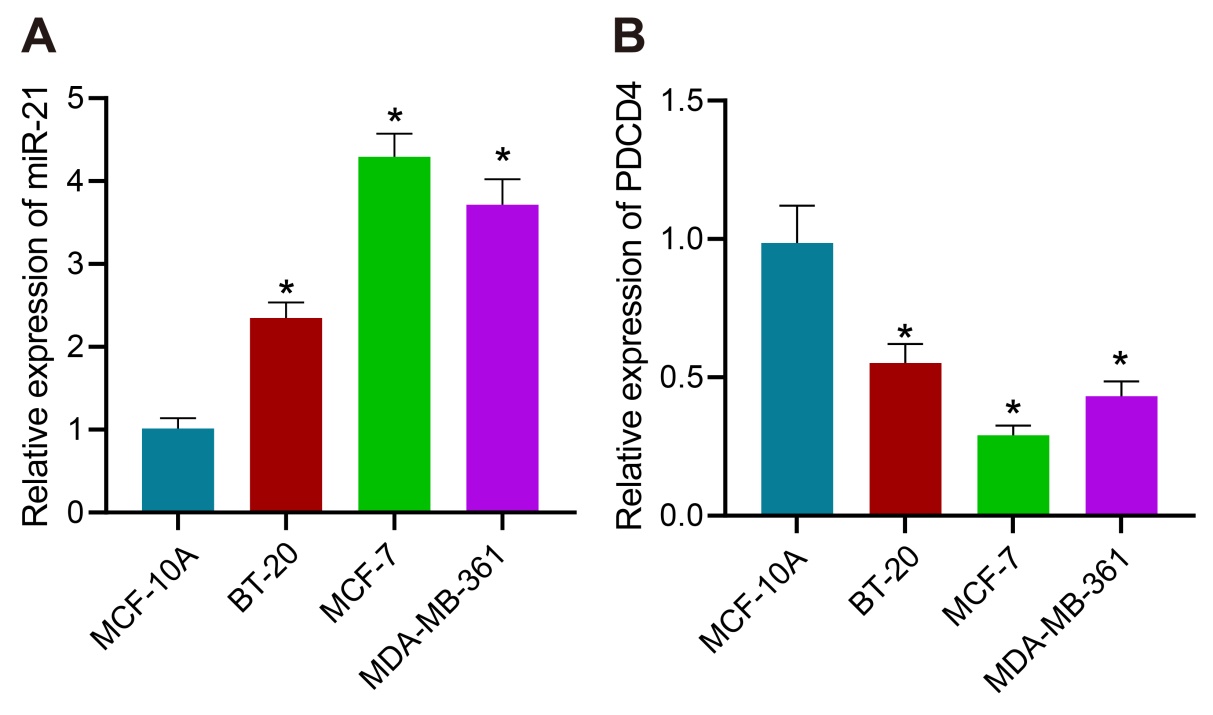
**

**FIGURE S2** Expression of miR-21 and PDCD4 in BC cells. A, miR-21 expression in human normal mammary epithelial cells (MCF-10A) and BC cells (BT-20, MCF-7, and MDA-MB-361) determined by RT-qPCR. B, PDCD4 expression in human normal mammary epithelial cells (MCF-10A) and BC cells (BT-20, MCF-7, and MDA-MB-361) determined by RT-qPCR. * *p* < 0.05 compared with MCF-10A cells. Data (mean ± standard deviation) among multiple groups were analyzed using one-way ANOVA. Each sample was evaluated three times independently.

**
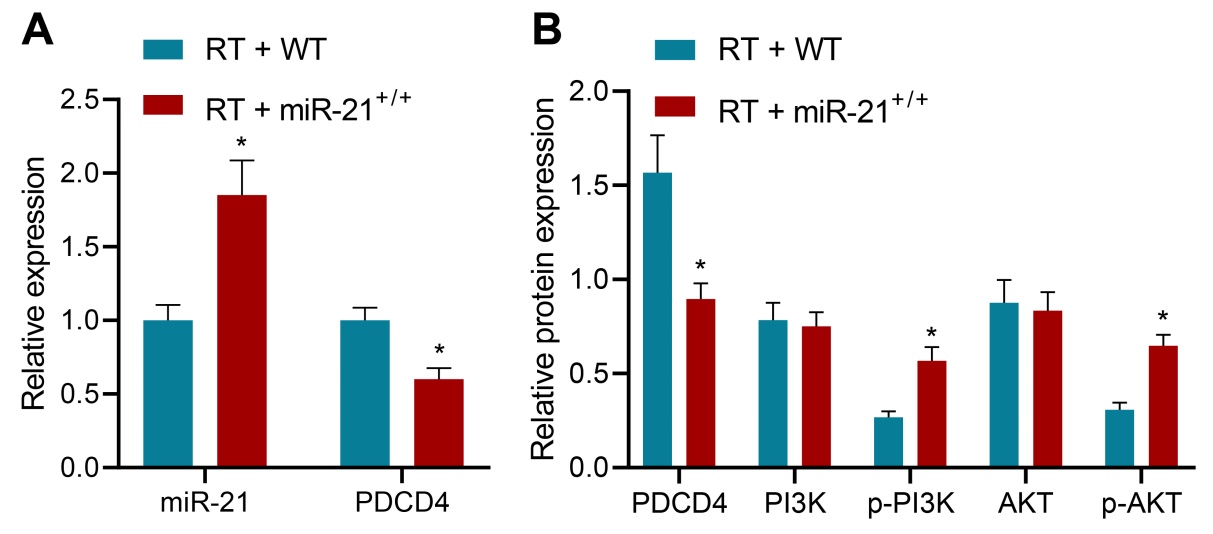
**

**FIGURE S3** miR-21, PDCD4, PI3K, and Akt expression in WT mice and miR-21^+/+^ mice after treatment with radiotherapy. A, miR-21 expression and PDCD4 mRNA expression in WT mice and miR-21^+/+^ mice after treatment with radiotherapy. B, PDCD4, PI3K, and Akt expression and PI3K and Akt phosphorylation levels in WT mice and miR-21^+/+^ mice after treatment with radiotherapy. * *p* < 0.05 compared with WT mice treated with radiotherapy. There were 10 mice in each group. Each sample was evaluated three times independently.

**
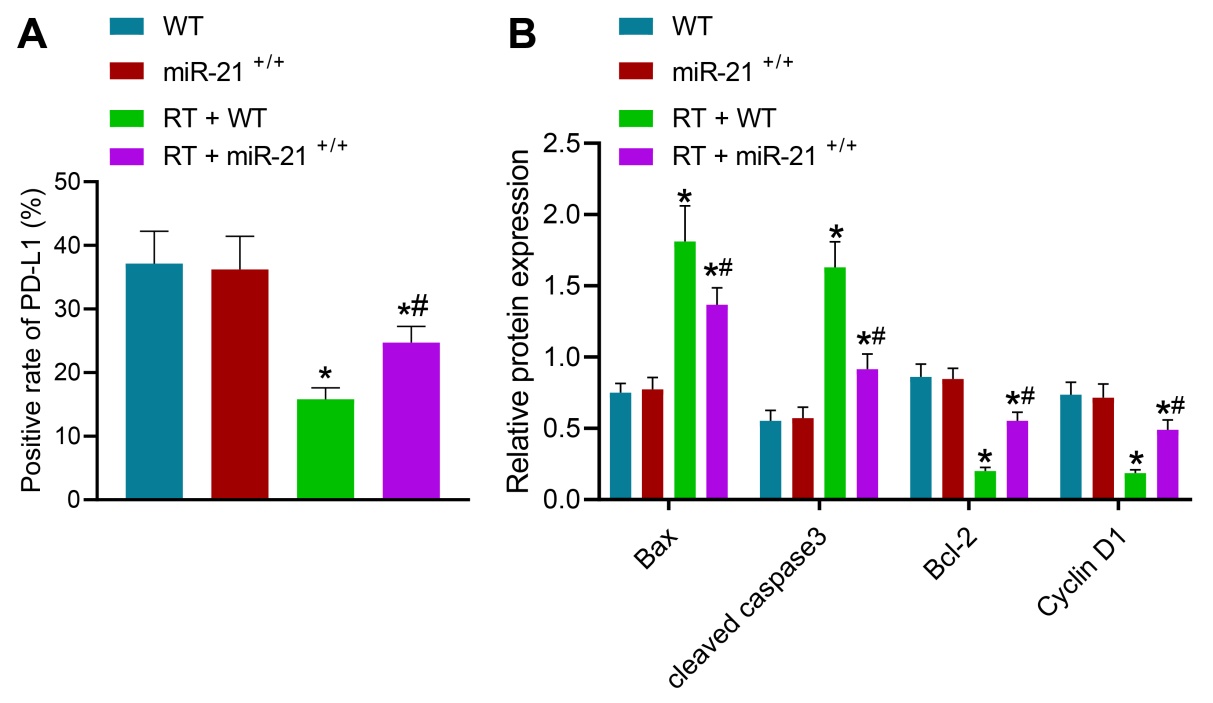
**

**FIGURE S4** Expression of PD-L1 and apoptosis-related factors in WT mice and miR-21^+/+^ mice treated with radiotherapy. A, PD-L1 positive cells in tumor tissues of WT mice and miR-21^+/+^ mice treated with radiotherapy measured by immunofluorescence. B, Protein levels of apoptosis-related factors in tumor tissues of WT mice and miR-21^+/+^ mice treated with radiotherapy determined by Western blot analysis. * *p* < 0.05, compared with WT mice treated with radiotherapy. There were 10 mice in each group.
